# Qualitative Assessment of Vaccine Hesitancy in Romania

**DOI:** 10.3390/medicina55060282

**Published:** 2019-06-17

**Authors:** David Miko, Carmen Costache, Horațiu Alexandru Colosi, Vlad Neculicioiu, Ioana Alina Colosi

**Affiliations:** 1 Iuliu Hațieganu University of Medicine and Pharmacy, 400349 Cluj-Napoca, Romania; dav.miko@gmail.com; 2 Department of Microbiology, Iuliu Hațieganu University of Medicine and Pharmacy, 400349 Cluj-Napoca, Romania; vlad.neculicioiu@gmail.com (V.N.); ioana.colosi@gmail.com (I.A.C.); 3 Department of Medical Education, Division of Medical Informatics and Biostatistics, Iuliu Hațieganu University of Medicine and Pharmacy, 400349 Cluj-Napoca, Romania; hcolosi@umfcluj.ro

**Keywords:** vaccine hesitancy, vaccine acceptance, MMR, HPV, qualitative thematic analysis, influence of media, measles outbreak

## Abstract

*Background and objectives:* Health systems all over the world are confronted with an alarming rise of cases in which individuals hesitate, delay, and even refuse vaccination, despite availability of quality vaccine services. In order to mitigate and combat this phenomenon, which are now defined by the World Health Organization (WHO) as vaccine hesitancy (VH), we must first understand the factors that lead to its occurrence in an era characterized by wide access to safe and effective vaccines. To achieve this, we conducted field testing of the Vaccine Hesitancy Scale (VHS), as it was developed by the Strategic Advisory Group of Experts Working Group (SAGE WG), in Cluj-Napoca city, Cluj County, Romania. The scale is designed to quantify VH prevalence in a population, establish which vaccines generate the highest percentage of hesitancy, and allow a qualitative assessment of the individual’s reasons for hesitance. *Materials and Methods:* We conducted an observational cross-sectional survey, which was comprised of descriptive, analytical, and qualitative elements regarding VH. The necessary sample size was 452 individuals. The VHS and Matrix of Determinants (recommended by SAGE WG) for reasons people gave to justify their hesitance, was interpreted by qualitative thematic analysis (QTA) to ensure the validity and reliability in detecting hesitancy across various cultural settings and permit global comparisons. *Results:* We found a VH of 30.3% and 11.7% of parents reported refusing to vaccinate their child. Among the VH responders, the varicella vaccine generated 35% hesitancy, measles vaccine 27.7%, Human Papillomavirus (HPV) 24.1%, and mumps vaccine 23.4%, respectively. The QTA values for percent agreement ranged from 91% to 100%. Cohen’s Kappa values ranged from 0.45 to 0.95. Contextual influences identified for VH were “media,” “leaders and lobbies,” and “perception of the pharmaceutical industry.” Individual and group influences for VH were “beliefs,” “knowledge,” and “risk/benefits (perceived).” Vaccine and vaccination specific issues for VH were “risk/benefit (rational)” and “health care practitioners (trustworthiness, competence).” *Conclusions:* One-third of the investigated population had expressed VH, and a further one-third of these had refused a vaccine for their child. Chicken Pox, Measles, Mumps, Rubella (MMR), and HPV vaccines generated the most hesitation. Negative information from the media was the most frequently evoked reason for VH.

## 1. Introduction

The World Health Organization (WHO) identifies vaccine hesitancy (VH) as one of the top 10 threats to global health [1]. Health systems all over the world experience a decrease in vaccine acceptance, which ultimately leads to outbreaks preventable by vaccination. In 2018, 10 countries in Europe had measles outbreaks (Ukraine, Serbia, Israel, France, Italy, Russian Federation, Georgia, Greece, Albania, and Romania) [2]. In April 2019, the state of New York declared a state of emergency in Rockland County by imposing a 30-day ban to unvaccinated children from public spaces and administered hundreds of doses of MMR vaccines during the crisis [3]. 

The scientific vaccination community constantly advises that, in order to promote vaccine acceptance, we should understand the factors that determine VH and tackle each of them individually. One particularity of vaccines is to serve several outcomes at once: avoiding disease at an individual level, while also protecting the community by preventing the spread of infectious diseases. The latter is referred to as “herd immunity,” and requires high vaccine uptakes in order to ensure efficient prevention against disease outbreaks [4]. Monitoring VH and its causes, which can lower herd immunity, has, therefore, become an indispensable part of the process, which leads to the implementation of adequate public health interventions [5,6].

Although access to health care has long been known to be a factor likely to influence vaccination rates, another important aspect to consider is the degree to which a population in question is open and, therefore, acceptant toward vaccination. Indecision and mistrust can hinder the effectiveness of immunization programs and, ultimately, lead to a decrease in vaccine uptake [7]. On that note, it is important to consider that concerns, skepticism, and misconceptions regarding vaccination have existed since its implementation when it was performed by pioneering doctors such as Zabdiel Boylston (1721) and Edward Jenner (1796–1798) [8,9].

Currently, the phenomenon of resistance and reluctance toward vaccines is facilitated, among other things, by the rising influence of the Internet [10], where all opinions can be “horizontally” shared, and all information, verified or not, is widely dispersed. Additionally, the Internet has the unique capacity to rapidly amplify the spread of information. This characteristic can become a challenging problem for public health forces when the information that is being shared is either unclear, in conflict with the scientific consensus, or simply erroneous [11]. 

One of the most spectacular examples of the devastating impact that false information can have on the public’s perception of vaccines was an article published in 1998, in the *Lancet*. The article in question presented false results that linked autism to the MMR vaccine. Although the scientific journal retracted the article 12 years later, many autism advocacy groups are still highly active on blogs and social media, defending a myth that has now long been debunked [12,13]. However, such myths are occasionally still fueled by highly influential and misinformed individuals, which further promotes their circulation. This represents a real problem in the realm of clinical medicine where information alone does not seem sufficient in convincing hesitant individuals to change their minds, and where clinicians often feel out of their depths in dealing with the rhetorical arguments [14].

Recognizing the importance of VH as a major public health issue, and its potential to impact vaccine coverage, the Strategic Advisory Group of Experts (SAGE) on Immunization established in March 2012 a Working Group (WG) that dealt with vaccine hesitancy. The terms of reference of SAGE WG are presented in the WHO report from November 2014 [4]. The SAGE WG on VH is assigned a number of missions. The first mission is to give a definition of hesitancy and its scope. The following definition is accepted.

*“Vaccine hesitancy refers to delay in acceptance or refusal of vaccination despite availability of vaccination services. Vaccine hesitancy is complex and context specific, varying across time, place, and vaccines. It is influenced by factors such as complacency, convenience, and confidence”*.

[6]

However, in 2015, an article featuring some of the members of the SAGE WG on VH attempted to bring some perspective and point out some of the limits of the vaccine hesitancy definition [15]. According to them, the concept was defined in ambiguous terms and established upon uncertain theoretical background. This, in turn, resulted in some studies using the term in ways that appeared at times contradictory, which jeopardized research and future interventions. They, therefore, proposed a different approach, which considers VH to be a decision-making process that depends on people’s level of commitment to healthism/risk culture (1st axis) and on their confidence in health authorities and mainstream medicine (2nd axis) [14]. 

A systematic review of 28 quantitative studies, conducted by Larson et al. (a member of the SAGE WG on VH) in 2018, notes that combined trust in the healthcare system, science, and government has an indirect effect on the likelihood of health care practitioners to recommend vaccination [16]. Moreover, their assessment of qualitative literature finds that a violation in trust was said to occur when health care practitioners, the government, or the wider health system were seen to financially profit from vaccination, which reminds us that vaccine-related trust exists within a context of deeper, underlying trust in society at large. 

The aim of our study is to assess the prevalence and particularities of VH in the population of parents in Cluj-Napoca city, Cluj County, Romania, and to uncover its underlying factors. 

## 2. Materials and Methods

This study focused on the qualitative analysis of factors underlying VH and the decrease in vaccine acceptance.

### 2.1. Study Design

The study was designed as an observational cross-sectional survey, which encompassed descriptive, quantitative, and qualitative analysis. 

The descriptive part aimed to establish the prevalence of vaccine hesitancy among the population of a medium-sized European city (Cluj-Napoca, Cluj County, Romania), while the analytical part strived to uncover possible links and factors that could explain its occurrence. The qualitative part was used as a mean to both enrich and refine the raw statistics with an outlook borrowed from social sciences and also assess the validity of the Matrix of Determinants (MxDt) for VH designed by SAGE, in accordance with their recommendations. 

### 2.2. Participants

A total number of 452 individuals were included in the study. Participants were recruited from a source population consisting of parents and/or guardians (hereafter referred to as parents) who accompanied their children to any of the four Pediatric Clinics in the city (Cluj-Napoca, Romania) for a consultation and/or hospitalization between the 1st of May and 30th of June 2018. 

The Raosoft sample size calculator (http://www.raosoft.com/samplesize.html) was used to ensure the recruitment of a sufficiently large number of participants in order to obtain statistically significant data, given the chosen of margin error (5%), confidence interval (95%), and approximate population size (300,000 individuals). 

### 2.3. Participant Recruitment and Informed Consent

Parents that were accompanying their children in the hospital were visited individually, in their respective rooms/waiting room, and invited to participate in the study. The researcher, after presenting himself and the purpose of the study, presented a brief overview of the VHS, and read out a consent form that explained what was required from them as participants. The participants were informed that participation was not compulsory and that the form was to be completed anonymously. Verbal consent was obtained every time, and willingness to fill in the questionnaire was taken as a further form of tacit consent.

Participants were administered the VHS questionnaire, designed by the SAGE WG on vaccine hesitancy in 2015 (Appendix A and Appendix B). The questionnaire was composed of three types of questions: core closed questions, Likert Scale questions, and open-ended questions [4,17,18]. The questionnaire was translated to Romanian for ease of access to participants and to eliminate any semantic confusion that might occur.

Once verbal consent had been obtained and the researcher had made sure that the participants had understood what was expected of them, they were left to answer the questions by themselves, in order to minimize the classification bias that could have stemmed from the presence of the researcher. On average, participants spent 30 min to one hour to complete the form.

### 2.4. Ethical Approval

This study was exempt from approval by an Ethics committee. 

The study was approved by the dean’s office of the University of Medicine and Pharmacy “Iuliu Hațieganu” Cluj-Napoca as a subject of graduation thesis (number 2258/7 Dec 2017).

The clinics where the questionnaires were distributed have an established agreement with the university through which all the data obtained from the patients may be used for a research purpose. 

Nevertheless, in our study, no medical or personal data of the participants were obtained, except of their own opinions expressed in the questionnaires. 

### 2.5. Data Analysis

The prevalence of hesitant individuals was determined by considering the frequencies of positive answers to core closed questions 3 and 4 (Appendix A), which include “Have you ever hesitated (…)” (question 3) or “(…) refused a vaccination for your child?” (question 4).

A qualitative thematic analysis (QTA) was performed to analyze participants’ answers to open questions. A deductive approach was chosen i.e., the codes and themes development were guided by existing concepts and ideas, including, in this instance, the SAGE WG Matrix of Determinants (MxDt) of vaccine hesitancy (Table 1). 

This method was also chosen for its convenience in terms of time, resources, and researchers’ limited experience in qualitative research [19]. According to the approach developed by Braun and Clarke [20], a combination of semantic and latent coding approaches was used to grasp both explicit and implicit content embedded within the data. 

The following steps were undertaken by two separate researchers.

1. Independent familiarization with the data set and MxDt of vaccine hesitancy.
2. This was followed by a meeting of the researchers in order to calibrate their understanding of how the 21 items of the SAGE MxDt was related to the dataset, which takes into consideration the variations of meaning implied by the wording of each question.
3. Preliminary training, which consists of a sample of 29 statements (4% of total 697 statements) was performed in order to calibrate the researcher’s understanding of how the data was related to the matrix and to minimize possible systematic gaps of interpretations (1 native researcher, 1 foreigner) as well as methodology described previously by Matthew Lombard [21]. This lasted for about 4 hours.
4. The two researchers then independently mapped the 697 statements directly against the 21 items of the Matrix, question by question, using a binary method (item present in the statement: 1/item absent from the statement: 0).
5. Inter-coder reliability, using “ReCal” [22], an online utility, compatible with Excel, that computes reliability coefficients for nominal, ordinal, or ratio-level data, was calculated for each of the 21 items, across the whole 697 statements, using percent agreement and Cohen’s Kappa [23]. Possible results for “percent agreement” are between 0 and 1, where “0” means that the authors who performed coding did not do better than if they had been working at random, while “1” means perfect agreement between the two raters. Negative results are possible, which means that the two authors did worse than if they would code accidentally. The cut-off values for Kappa were determined according to the methodology described by Hallgreen [24] and Krippendorff [25].
  - from 0.0 to 0.2 indicating slight agreement
  - 0.21 to 0.40 indicating fair agreement
  - 0.41 to 0.60 indicating moderate agreement
  - 0.61 to 0.80 indicating substantial agreement
  - 0.81 to 1.0 indicating almost perfect or perfect agreement

The deductive approach to the thematic analysis of the qualitative content expressed by the participants enabled the researchers, in the manner that Dubé et al. investigated immunization managers opinion on vaccine hesitancy [26], to test whether the SAGE WG Matrix of Determinants of vaccine hesitancy (Table 1) was relevant in the context of grasping people’s reasons for hesitancy in the setting of a middle-sized city from Eastern Europe (WHO EUR region). This task was performed in accordance with the recommendation of SAGE, who recommended having their work on vaccine hesitancy field-tested to ensure that it was relevant worldwide [4,18].

Besides the already mentioned methods used for sample size calculation, qualitative thematic analysis (QTA), and computation of inter-coder reliability, quantitative data has also been collected, described, and analyzed using Microsoft Excel 2010, BiostaTGV [27], and VassarStats: Website for Statistical Computation [28]. 

## 3. Results

### 3.1. Brief Overview of Descriptive and Quantitative Results

The prevalence of vaccine hesitant individuals, defined as a positive answer in either question 3 (hesitancy) or question 4 (refusal) was 30.3% (95% CI 26.3–34.7 %), which corresponded to 137 out of 452 responders. 

The varicella vaccine generated the most reluctance among hesitant individuals, 35% (95% CI 27.6–43.3%), but other vaccines also scored reluctance rates above 20% among vaccine hesitant respondents: Measles vaccines, 27.7% (95% CI 20.9–35.8%), HPV vaccine, 24.1% (95% CI 17.7–31.9%), Mumps vaccine, 23.4% (95% CI 17.1–31.1%), and Rotavirus vaccine, 22.6% (95% CI 16.4–30.3%).

The prevalence of those who actually refused a vaccination (48 responders) among those who also declared being hesitant regarding vaccines (133 responders) was 36.1% (95% CI 28.4–44.5 %).

### 3.2. Qualitative Thematic Analysis (QTA)

The inter-rater reliability measures computed for each item of the MxDt are presented in Table 2.

Items 5, 6, 8, and 18 reaped a score below 0.8 (0.60, 0.67, 0.54, and 0.46, respectively). It is worth noting that these items were among those which came up the least, which also explains why each disagreement between the researchers weighed more. It is possible that this phenomenon can be overcome by increasing the sample size, with the condition to have a more frequent repetition of these items by the responders. Additionally, items 3 and 17 were not mentioned once by either researcher, while those statements labelled “not codable” (*n* = 17) reflected comments such as “I do not know” rather than specific ideas outside the matrix. The coding guide is found in Appendix C. 

Table 3 summarizes how participants’ answers to the open questions of the VHS are related to the determinants of vaccine hesitancy in the model developed by SAGE WG.

#### 3.2.1. Contextual Influences

##### Item 1 “Media”

This item was overwhelmingly evoked by participants to explain the growing reluctance to accept vaccines. Of note here, is the role of Internet and the emergent role of social media such as Facebook, in particular, for its propension to share anecdotal experiences, anti-vaccines campaigns, and science denigrating content. This appears to be acknowledged across the narratives of both hesitant and non-hesitant participants: participant 341 “*Facebook,*” participant 314 “*too much access to wrong information (Internet),*” participant 332 “*the media is presenting positive and negative information and sadly a trend to not vaccinate the children emerged,*” participant 405 “*because, in the media and social networks, the adverse effects of vaccines are being highlighted to the detriments of their benefits.*” Nonetheless, it should be remembered that more traditional media such as television were also incriminated (participant 173 “*I’ve seen on TV that vaccines are not indicated,*” and participant 430 “*I have seen on TV some parents who refuse to vaccinate their children.*”)

##### Item 2 “Leaders and Lobbies”

This idea was frequently raised explicitly by participants (participant 35 “they give too much credit to non-medical (unqualified) sources,” participant 94 “*they are being manipulated by badly informed persons with regards to the long-term benefits of vaccines,*” participant 213 “*accepting opinions from people who have no medical background and have no idea of medical progress,*” and participant 214 “*a big pressure is put on this topic.*” 

However, the role of anti-vaccination lobbies was most frequently reflected indirectly or implicitly in the discourse, by the negative undertone associated with specific or elaborated anecdotes. These language elements were interpreted as illustrating, to varying degrees, the perception of the influence of parties following a certain agenda. Item 1 and 2 were, therefore, frequently associated in those cases, which seemed coherent, since media, especially the Internet, represent channels of choice for lobbies to spread their ideas: participant 416 “*they heard about cases in which children have died and they believe that to be caused by vaccines,*” participant 414 “*because of stories they heard in the media regarding adverse effects, with sick children because of the vaccines,*” participant 329 “*because of theories that say that you should not vaccinate your children because of their adverse effects are gaining weight, and they manage to influence the parents,*” and participant 163 “*viral online campaigns against vaccination, which, even though they will not convince someone, will generate suspicion and confusion*.”

##### Item 7 “Perception of the Pharmaceutical Industry”

Whether vaccine hesitant or not, participants expressed concerns relating to conflicts of interests and the ethics of pharmaceutical companies: the appeal for financial gain was often perceived as superseding the public health interests of the population, participant 145 “*lack of trust in the pharmaceutical companies,*” participant 38 “*vaccines manufacturers are not held responsible for the adverse effects,*” participant 200 “*I cannot be sure vaccination puts the health of my child as the top priority,*” participant 410 “*in general, parents are afraid that vaccines are below required standards and that they could be counterfeit. Although they consider vaccines important, they do not trust the institutions that manufacture the vaccines*.”

More specifically relating to Romania, some people also expressed doubts regarding the products they had access to in their part of the world: participant 209 “*low-quality products that do not respect European standards.*”

#### 3.2.2. Individual and Group Influences

##### Item 9 “Beliefs”

This was encountered in both explicit and latent form. Although some data pointed towards specific beliefs in alternate narratives than that of science, much of this code was encountered as a way of expressing an irrational fear of adverse reactions: participant 424 “*personal superstitions,*” participant 137 “*they believe themselves to be emancipated, ecologist, saviors of the world,*” participant 449 “*fear that vaccines can cause more harm than good,*” and participant 400 “*the refusal of many people appears out of fear of possible adverse reactions or because they do not believe in their efficiency.*”

##### Item 10 “Knowledge” 

This item was also very common, especially among those who were not vaccine hesitant, as a mean to rationalize the choices and attitudes of vaccine hesitant people. “Knowledge” was understood both as facts and data one needs to understand a particular phenomenon, as well as the critical thinking needs in order to go through a decision-making process (participant 231 “*Ignorance,*” participant 67 “*they do not understand that it is necessary, they are not correctly informed,*” participant 277 “*lack of scientific information explained for the layman,*” participant 145 “*negative information heard about vaccines without putting information within proper context,*” participant 214 “*every parent is bombarded with information, which is either pro or anti vaccine, and sometimes they cannot distinguish between what is good and what is harmful to their children,*” and participant 349 “*Some people can be influenced very easily. They cannot discriminate between relevant and irrelevant information*.”).

##### Item 12 “Risk/Benefits (Perceived)”

This item was interpreted as a very common latent element throughout the data especially where answers related to risk assessment as a rather intuitive and emotional process rather than relying on rationality and logic (participant 61 “*they have unwanted effects, are dangerous, and can determine serious diseases,*” participant 204 “*from the information that I have, the number of cases that presents adverse effects from vaccines is approximatively the same as the cases where children get sick without vaccination and, therefore, I believe that every parent has the right to choose if they want to vaccinate or not,*” participant 23 “*fear of adverse reactions,*” participant 110 “*they do not know the substances enough,*” and participant 163 “*Maybe there are too many vaccines?*”).

#### 3.2.3. Vaccine and Vaccination Specific Issues

##### Item 14 “Risk/Benefit (Rational)”

This item was the most often raised item and this is because, just as item 12, it was often identified as a latent code in the data, with the additional nuance of rationality (or lack of it) as opposed to item 12, which is related more to a heuristic model of risk/benefit assessment. In that sense, this code was also perceived as underlying the rationalizing process of non-hesitant parents to explain the attitudes of hesitant ones (participants 418 “*they cannot understand what could happen to their children,*” participant 437 “*misinformation regarding what vaccines mean for the good health of the child,*” participant 396 “*the incapacity to understand what is better for the child,*” participant 344 “*They are getting their information from uncertain sources (Internet). Their decision to refuse vaccination is unfounded*.”). Nonetheless, some people did root their hesitation in rational elaborations (participant 348 “*in my opinion, the vaccines have not been studied well enough,*” participant 365 “*if vaccines have not been sufficiently tested or if they are not suitable for the zone in which we live,*” participant 358 “*some new vaccines should be tested for a longer period of time so the long term adverse effects are better understood*.”).

##### Item 21 “Health Care Practitioners”

This item was encountered in both explicit statements relating to attitudes and latent ones related to the trustworthiness of the information received. Some explicit statements pointed to the responsibility of doctors (and general practitioners, in particular) in their influencing role on vaccine issues (participant 174 *“the former family doctor was anti-vaccine,”* participant 408 “*Sometimes they are misinformed. Other times the GPs are skeptical with regards to vaccines.*”) Others suggested a general distrust targeted at the practitioner’s reliability and competence (participant 5 “*Some GPs do not store the vaccines in good conditions,*” “*lack of information of doctors about the vaccines he administers,*” participant 162 “*fear from adverse reactions and vaccination without proper exams/investigations,*” participant 119 “*because some vaccines and needles are not sterile and, therefore, some ugly viruses could be spread from a patient to another. I would like to see more attention and competence in this setting,*” and participant 134 “*lack of trust in the medical system*.”).

## 4. Discussion

Over the past decade, Romania has witnessed a constant decrease in immunization coverage despite optimization of vaccination campaigns and vaccine supply availability. Measles and mumps (ROR vaccine) vaccination uptake plummeted from 95% in 2010 to 87% (for dose I) and 74.7% (dose II) in 2017. These percentages illustrate a precarious situation. In 2017, Romania became the country with the highest number of cases among all EU nations (with 3072 cases of measles reported between April 2016 and April 2017) [29]. Romania serves as a prime example of the devastating effect that a decrease in vaccine uptake can have on the wellbeing of a nation and, more so, on the importance of efficient and prompt identification of the factors that lead to vaccine hesitancy within the population.

It has been pointed out for some time now that the Internet has become a major source of access to health-related content [30], as well as a tool for activists in their campaigns against the scientific establishment [6,31]. The role of social media, in particular, has been shown [32,33] and researchers encourage the input of social science research and social movement analysis, in particular, to aid the dynamics of vaccination criticism [34]. Semantic research into Internet content has also indicated that future public health efforts need not only focus on the benefits of vaccination but, more so, on understanding the nature of the doubts at the core of the vaccine hesitancy issue in order to improve communication strategies [35,36].

The present study did not identify religion as a hindrance to vaccine acceptance in the population studied. This is contradictory with other papers on the topic, which had identified religion as a major predictor of negative attitudes toward science and science literacy [37]. 

Where beliefs are concerned, comments from the participants mirrored well the common tropes already identified by 2012 in a study of the rhetorical tactics used by anti-vaccine activists on Internet, which include “*I’m not anti-vaccine, I’m pro-safe vaccines,*” “*Vaccines are toxic!”, “You cannot prove vaccines are safe,” “Vaccines are unnatural,” “You are in the pocket of Big Pharma,” and “I am an expert on my own child*” [38]. It has been proposed that humans cope with risk in three fundamental ways: risk as feelings (represented by our intuitive and fast reactions to danger), risk as analysis (which brings logic, reasons, and slow deliberation in the process of hazard management), and, lastly, risk as politics [39]. Our propension to predominantly deal with “risk” with our feelings can, perhaps, explain why items on ’’risk/benefits’’ (as heuristics or rationality) appeared so often in our analysis. 

Even where rationality is concerned, as Damasio points out, “*it is unlikely that we can employ analytic thinking rationally without guidance from affect somewhere along the line*” [40]. These views are supported by other risk perception research, which identified several elements pertaining to the fear of vaccines. These elements are: 1. risks to children evoke more fear than those to adults, 2. risks are weighted against benefits and the benefit of vaccines has gone down precisely because they have been so effective, 3. man-made risks worry us more than natural ones, 4. the less control we feel over a specific risk, the more worried we are likely to be, and, lastly, 5. we are more worried about risks produced by people or institutions we do not trust [41]. 

Some of our participants also reported negative or insufficiently encouraging attitudes of doctors. Vaccine hesitancy among healthcare workers in Europe has been reported in many studies [42,43,44,45] and constitutes a real source of worry since the role of healthcare workers is known to be primordial in the pedagogy of vaccine acceptance [46]. Some studies have suggested that more efforts made to communicate the scientific and medical consensus around childhood vaccines tend to increase public support of vaccination [47]. Other studies in Romania have shown that increased knowledge among medical students was correlated with increased vaccine acceptance, which highlights the need for the relevant information to be learned by representatives of the medical profession as essential elements of public health strategies [48]. 

It should be noted, however, that scientific knowledge is not the only skill identified for influencing behavior related to vaccines. One study found that health care providers are often inadequately trained to manage difficult conversations with reluctant parents and identified this lack of preparation as an inhibiting factor for recommending vaccination [49]. 

Based on our results, we suggest the refinement of the item 1 (communication & media environment) of MxDt, which was associated with the second highest number of iterations—314. One possible idea for refinement of this item might be the introduction in the matrix of an item related to trendsetters. In our study, item 3 (Historical influences) and item 17 (design of vaccination program) do not cover any iteration from the responders, while item 6 (geographical barriers) cover only two iterations. This might be a signal for a modification or even elimination of this determinant from the matrix. Items 15, 18, and 20 were also associated with a very low number of iterations—seven, which might be suggestive for the need to improve the communication of the medical information to the public, because all of these items belong to the vaccine/vaccination-specific issues category.

In order to increase vaccination acceptance, some authors suggest that maternal vaccination should be more widely publicized, which gives the sensation that care is shared rather than individualized [50]. This approach, and also the strategies implemented to improve mothers’ experiences when their newborn is vaccinated, would most likely prevent the development of VH toward vaccination of their children [51]. To promote vaccine acceptance, materials with positively-framed messages (e.g., “vaccines keep you healthy”, “no vaccine-preventable diseases kill you”) may be used, either like infographics or videos. In a qualitative study of mothers who are hesitant about vaccines, authors found that short videos were received more favorably than the infographics, with most participants stating that videos did a better job of showing the concepts because of the use of animation, colors, sounds, and familiar analogies [52]. Another study, made in US and Australia, emphasize the importance of pro-vaccine parent blogs and discussion groups pushing for policy change rather than public confrontation [53].

### 4.1. Study Strengths

To our knowledge, this study is the first to ever use the tools recently developed by the WHO to monitor vaccine hesitancy in Romania. It will bring a valuable contribution to public health strategies, particularly regarding Tailoring Immunization programs (TIP), which is a guide developed by WHO to help national immunization programs (NIPs) design targeted strategies to increase uptake of infant and childhood vaccinations [26,54].

### 4.2. Study Limitations

#### 4.2.1. Selection Bias

In an overwhelming majority (80%), the parent was the mother. Female gender is, therefore, over-represented in our sample.

The use of a written questionnaire, that had to be filled entirely by the participants himself, also determined another bias: a large proportion of the Roma population (albeit not its totality) was not involved in the study, due to outright refusal to participate or due to a lack of literacy. Literacy issues have been identified in previous studies as a barrier to health care access in the Roma community, and a contributing factor to them perceiving the health institutions as a threat, which hinders their compliance to treatments [55,56]. Collectivism plays a large part in Roma individuals’ sense of self and identity. Therefore, the weight of the community in the individual’s decision-making process can be misunderstood, which decreases the potential for effective interventions of health care professionals [49]. This issue is particularly important since unvaccinated or under-vaccinated individuals tend to cluster together in specific geographic locations and constitute possible vectors of propagation of infectious diseases, which undermines public health efforts in countries where the rates of vaccine uptake in the rest of the population remains substantially high [5,41,57,58,59].

#### 4.2.2. The Questions Were Not Pilot-Tested

There was no pilot-testing of the questions, as recommended in SAGE’s report from 2014 [4]. A pilot test could have helped the researchers reflect and alter the question sequence, in order to maximize answer accuracy [4,17]. It seems plausible that the open answers (presented last in the questionnaire) could have been influenced by the preceding content.

#### 4.2.3. Use of Thematic Analysis vs. Computer Software

Deductive thematic analysis was used to determine whether the statements of the respondents fitted the SAGE WG MxDt of vaccine hesitancy, as opposed to using an electronic data extraction form whose data would then be mapped against the Matrix, such as in the study of Dubé et al. [60]. 

It is now well understood that, for a society to successfully achieve high vaccination coverage with a high vaccine acceptance, which represents a public health goal of paramount importance both from a humane and an economic point of view, its population needs to first and foremost have a rational understanding of its benefits [61]. As Obregan et al. (2009), cited by Goldstein et al., wittingly puts it: “*There is no vaccine against resistance or refusals that are rooted in social, cultural, religious, and political contexts*” [62]. 

Further work is, therefore, required, both to fill in the gaps from this study and to keep track of the phenomenon, which is, by definition, evolving over time. This, in turn, should give public health authorities the best chance to design adequate policies to face the unveiled factors of VH within the study area, for better vaccine acceptance.

## 5. Conclusions

1. The prevalence of vaccine hesitant individuals in our study was 30.3%.
2. The prevalence of those who refused a vaccination among hesitant individuals was found to be 36.1%.
3. The chickenpox (varicella) vaccine generated the most reluctance among hesitant individuals (35%).
4. The following vaccines scored above 20% in frequency among vaccine-hesitant participants: measles vaccines (28%), HPV vaccine (24%), mumps vaccine (23%), and rotavirus vaccine (23%).
5. Contextual influences identified for VH were “media,” “leaders and lobbies,” and “perception of the pharmaceutical industry.”
6. Individual and group influences for VH were “beliefs,” “knowledge,” and “risk/benefits (perceived).”
7. Vaccine and vaccination specific issues for VH were “risk/benefit (rational),” and “health care practitioners” (trustworthiness, competence).

## Figures and Tables

**Table 1:** Matrix of Determinants (MxDt) of vaccine hesitancy (VH).

**Contextual influences**	Communication & media environment Influential leaders, immune Historical influences Religion/culture/gender/socio-economic Politics/policies Geographical barriers Perception of pharmaceutical industry
**Individual and group influences**	8. Personal, family, and/or community member experience with vaccination, including pain 9. Beliefs, attitudes 10. Knowledge/awareness 11. Health system and providers-trust and personal experience 12. Risk/benefits (perceived, heuristics) 13. Immunization as a social norm
**Vaccine/vaccination specific issues**	14. Risk/benefits (epidemiological and scientific evidence) 15. Introduction of a new vaccine 16. Mode of administration 17. Design of vaccination program 18. Reliability and/or source of vaccine 19. Vaccination schedule 20. Costs 21. Strength of recommendation

**Table 2:** Inter-rater reliability measures for the items of the MxDt of VH.

Item	Percent Agreement	Scott’s Pi	Cohen’s Kappa	Krippendorff’s Alpha	No. Agreements	No. Disagreements
**Item 1**	94.83	0.90	0.90	0.90	660	36
**Item 2**	95.55	0.88	0.88	0.88	665	31
**Item 3**	100.00	1	1	1	696	0
**Item 4**	99.71	0.95	0.95	0.95	694	2
**Item 5**	97.84	0.60	0.60	0.60	681	15
**Item 6**	99.86	0.67	0.67	0.67	695	1
**Item 7**	92.96	0.81	0.81	0.81	647	49
**Item 8**	97.41	0.54	0.54	0.54	678	18
**Item 9**	94.54	0.87	0.87	0.88	658	38
**Item 10**	94.40	0.89	0.89	0.89	657	39
**Item 11**	96.12	0.79	0.79	0.79	669	27
**Item 12**	89.51	0.79	0.79	0.79	623	73
**Item 13**	98.13	0.86	0.86	0.86	683	13
**Item 14**	91.81	0.81	0.81	0.81	639	57
**Item 15**	99.71	0.83	0.83	0.83	694	2
**Item 16**	99.57	0.85	0.85	0.86	693	3
**Item 17**	100.00	1	1	1	696	0
**Item 18**	98.99	0.46	0.46	0.46	689	7
**Item 19**	98.71	0.85	0.85	0.85	687	9
**Item 20**	99.71	0.86	0.86	0.86	694	2
**Item 21**	96.12	0.84	0.84	0.84	669	27
**Item 22**	99.14	0.83	0.83	0.83	690	6

**Table 3:** Deductive Thematic Analysis.

SAGE WG Vaccine Hesitancy Model *					
Contextual Influences		Individual and Group Influences		Vaccine and Vaccination-Specific Issues	
Items	Iterations	Items	Iterations	Items	Iterations
1	314	8	27	14	477
2	165	9	225	15	7
3	0	10	303	16	10
4	22	11	68	17	0
5	23	12	377	18	7
6	2	13	46	19	31
7	177			20	7
				21	101
TOTAL (%)	703 (29.2)	TOTAL (%)	1046 (43.5)	TOTAL (%)	640 (26.6)

* Total statements: 697. Statements considered “not codable”: 17. SAGE WG: Strategic Advisory Group of Experts Working Group.

## Appendix A

Vaccine hesitancy scale as designed by SAGE WG [4].

## Appendix B

Vaccine hesitancy open ended survey questions

## Appendix C

Coding guide used by researchers for 21 items of the MxDt of VH.

**Questions 6, 7, and 8+9 from VHS (Appendix A)**

**Question 6: Have any factors (distance, time necessary to wait, costs to get to the clinic, etc.) contributed to not vaccinate your child?**

❖ adverse reactions/side effects/any answer (specific or not) describing an adverse reaction
  - with no additional statement i.e., “adverse reaction”/”side effects” type of answer -> items 12 +14
  - if word “fear” is added (“fear of…”) -> item 9 + 12 + 14
  - specific or unspecific statement of a serious adverse reaction (including: “fake news” type and “absurd/obscure” and “serious” type) -> item 1 + 7 + 9 + 10 + 12 + 14
  - specific believable/possible statements (ex: fever/allergy/jaundice) -> item 10 + 12 + 14
❖ vaccine
  - distance -> item 6 + 20
  - lack of availability -> item 18 + 20
❖ personal health issue -> requiring delay or refusal in vaccination -> item 19
❖ trusts/distrust statements
  - without additional precision -> item 7 + 11
  - about the health care personal -> item 7 + 11 + 21
  - health system -> item 5 + 7 + 11 + 21 (+ item 1 if aberrant/absurd/conspiracy-like tone)
  - consent -> item 7 + 11 + 21
❖ information (info)
  - lack of info -> item 10 + 14 + 21
  ▪ lack info about healthcare system/manufacturer -> item 7 + 14 + 21
  ▪ lack info about effects/adverse reaction -> item 7 + 12 + 14 + 21
  - bad info/misinformation -> item 1 + 2 + 10
  - failure to inform oneself -> item 10 + 13 + 14
  ❖ health care personal attitude related statements -> item 21
  ❖ community participations/norms (nursery) -> item 5 + 13

**Question 7: Are there any reasons/pressures in your life that prevent you from vaccinating your child?**

❖ adverse reactions/side effects/any answer (specific or not) describing an adverse reaction
- with no additional statement i.e., “adverse reaction”/”side effects” type of answer -> item 12 +14
  ▪ if word “fear” is added (“fear of…”) -> item 9 + 12 + 14
- specific or unspecific statement of serious adverse reaction (including: “fake news” type and “absurd/obscure” and “serious adverse reactions” type) -> item 1 + 7 + 9 + 10 + 12 + 14
- specific believable/possible statements (ex: fever/allergy/jaundice) -> item 10 + 12 + 14
  ❖ health care personal attitude statement -> item 21
  ❖ vaccine
- content -> item 14
- administration -> item 14 + 16
- safety (vaccine themselves/production process) -> item 12 + 14
- efficiency -> item 14
- timing -> item 19
- new/old -> item 15
- cost -> item 20
- aberrant statement about vaccines effects -> item 1 + 14
- vaccine associated with statement of trust/uncertainty -> item 11 + 14
- vaccine + statement of necessity/no necessity (and, therefore, underlying idea of norm) -> item 13 + 14
❖ personal health issue -> requiring delay or refusal in vaccination -> item 19
❖ religion -> item 4
❖ trusts/distrust statements
- without additional precision -> item 7 + 11
- about the health care personal -> item 7 + 11 + 21
- health system -> item 5 + 7 + 11 + 21 (+ item 1 if aberrant/absurd/conspiracy-like tone)
- consent -> item 7 + 11 + 21
❖ media -> item 1
❖ perception health system (interest, intentions, hidden or not) -> item 2 + 7
❖ information
- lack of info -> item 10 + 14 + 21
  ▪ lack info about healthcare system/manufacturer -> item 7 + 14 + 21
  ▪ lack info about effects/adverse reaction -> item 7 + 12 + 14 + 21
- inferior information/misinformation -> item 1 + 2 + 10
- failure to inform oneself -> item 10 + 13 + 14

**Question 8+9: Are there any reasons for which you can think of why children should not be vaccinated/Have you ever heard negative information about vaccines? (because of the wording of the question, no answer can be understood "a priori" as the respondent opinion unless clearly stated)**

❖ “adverse reactions”/“side effects”
- with no additional explanation -> item 1 +14
- specific or unspecific statement of serious adverse reaction (including: “fake news” type and “absurd/obscure/unclear” type) -> item 1 + 2 + 7 + 9 + 10 + 12 + 14
- specific statements about believable/true statements -> item 1 + 14
❖ information
- negative information received through friends/family/community -> item 8 + 12 + 14
- negative information from media/unspecific statement -> item 1 + 12 + 14
❖ media -> item 1
❖ campaigns/lobbies -> item 1 + 2
❖ trusts/distrust statements
- without additional precision -> item 1 + 7 + 11
- vaccines as a business -> item 1 + 2 + 7 + 11
❖ vaccine
- safety (content/effects/manufacturing process) -> item 1 + 14 + 12
- vaccine + statement of necessity/no necessity (and, therefore, underlying idea of norm) -> item 1 + 13 + 14
- efficiency -> item 1 + 14
- new/old -> item 1 + 15
❖ health care personal attitude statement -> item 21

**Open questions from Appendix B**

**OPEN Q. 2: Do you have any worries about taking your child to vaccinate?**

❖ adverse reactions/side effects/any answer (specific or not) describing an adverse reaction
- with no additional statement i.e., “adverse reaction”/”side effects” type of answer -> item 12 +14
- if word “fear” is added (“fear of…”) -> item 9 + 12 + 14
- specific or unspecific statement of serious adverse reaction (including: “fake news” type and “absurd/obscure” and “serious adverse reactions” type) -> item 1 + 7 + 9 + 10 + 12 + 14
- specific believable/possible statements (ex: fever/allergy/jaundice) -> item 10 + 12 + 14
❖ vaccines themselves
- content -> item 14
- safety -> item 12 + 14
- efficiency -> item 14
- source of vaccine -> item 18
- administration -> item 16
- pain + vaccination -> item 8 + 16
- timing -> item 19
- new vs. old vaccine -> item 15
- vaccine + statement of necessity/no necessity (and, therefore, underlying idea of norm) -> item 13 + 14
- wrong belief (specific statement about mechanism or consequences that are wrong/not proven) -> item 9 + 10 + 14
❖ trusts/distrust statements
- without additional precision -> item 7 + 11
- about the health care personal -> item 7 + 11 + 21
- health system -> item 5 + 7 + 11 + 21 (+ item 1 if aberrant/absurd/conspiracy-like tone)
- consent -> item 7 + 11 + 21
❖ pharma industry/medical system/healthcare system -> item 7 + item 11
❖ responsibility for negative consequences -> item 5 + 7 + 11
❖ media (all types) -> item 1
❖ personal reasons/personal beliefs -> item 9 + 11
❖ personal health issue -> item 19
❖ information
- lack of information -> item 10 + 14 + 21
▪ about healthcare system/manufacturer -> item 7 + 14 + 21
▪ about effects/adverse reactions -> item 7 + 12 + 14 + 21
- inferior information/misinformation -> item 1 + 2 + 10
- failure to inform oneself -> item 10 + 13 + 14
❖ health care personal attitudes -> item 21

**OPEN Q. 4: If you refused immunization last year: why?**

❖ adverse reactions/side effects/any answer (specific or not) describing an adverse reaction
- with no additional statement i.e., “adverse reaction”/ “side effects” type of answer -> item 12 +14
▪ if word “fear” is added (“fear of…”) -> item 9 + 12 + 14
- specific or unspecific statement of serious adverse reaction (including: “fake news” type and “absurd/obscure” and “serious adverse reactions” type) -> item 1 + 7 + 9 + 10 + 12 + 14
- specific believable/possible statements (ex: fever/allergy/jaundice) -> item 10 + 12 + 14
❖ health care personal attitude statement -> item 21
❖ vaccine
- content -> item 14
- administration -> item 14 + 16
- safety (of vaccine themselves/of production process) -> item 12 + 14
- efficiency -> item 14
- timing -> item 19
- new/old -> item 15
- cost -> item 20
- aberrant statement about vaccines effects -> item 1 + 14
- vaccine + statement of trust/uncertainty -> item 11 + 14
- vaccine + statement of necessity/no necessity (and, therefore, an underlying idea of norm) -> item 13 + 14
❖ personal health issue
❖ requiring delay or refusal in vaccination -> item 19
❖ religion -> item 4
❖ trusts/distrust statements
- without additional precision -> item 7 + 11
- about the health care personal -> item 7 + 11 + 21
- health system -> item 5 + 7 + 11 + 21 (+ item 1 if aberrant/absurd/conspiracy-like tone)
- consent -> item 7 + 11 + 21
❖ fear -> item 9

**OPEN Q. 5: In your opinion, why do people refuse vaccines?**

❖ adverse reactions/side effects/any answer (specific or not) describing an adverse reaction
- with no additional statement i.e., “adverse reaction”/”side effects” type of answer -> item 12 +14
▪ if word “fear” is added (“fear of…”) -> item 9 + 12 + 14
- specific or unspecific statement of serious adverse reaction (including: “fake news” type and “absurd/obscure” and “serious adverse reactions” type) -> item 1 + 7 + 9 + 10 + 12 + 14
- specific believable/possible statements (ex: fever/allergy/jaundice) -> item 10 + 12 + 14
❖ vaccine
- content -> item 14
- administration -> item 14 + 16
- safety (of vaccine themselves/of production process) -> item 12 + 14
- efficiency -> item 14
- timing -> item 19
- new/old -> item 15
- cost -> item 20
- aberrant/absurd statement about vaccines effects/mechanism -> item 1 + 14
- vaccine + statement of trust/uncertainty -> item 11 + 14
- vaccine + statement of necessity/no necessity (and, therefore, underlying idea of norm) -> item 13 + 14
❖ trusts/distrust statements
- without additional precision -> item 7 + 11
- about the health care personal -> item 7 + 11 + 21
- health system -> item 5 + 7 + 11 + 21 (+ item 1 if aberrant/absurd/conspiracy-like tone)
- consent -> item 7 + 11 + 21
❖ information
- lack of information -> item 10 + 14 + 21
▪ lack information about healthcare system/manufacturer -> item 7 + 14 + 21
▪ lack information about effects/adverse reactions -> item 7 + 12 + 14 + 21
- inferior information/misinformation -> item 1 + 2 + 10
- failure to inform oneself -> item 10 + 13 + 14
❖ responsibility for negative consequences -> item 5 + 7 + 11
❖ media (all types) -> item 1
❖ personal reasons/personal beliefs -> item 9 + 11
❖ personal health issue -> item 19
❖ “recklessness”/«carelessness»/moral judgment (“doing wrong”) -> item 9 + 10 + 12 + 13
❖ education level/understanding -> item 10
❖ “fear” -> if without any other precision -> item 7 + 9 + 12
❖ health care personal attitude statement -> item 21

## References

[1] World health Organization Ten Threats to Global Health in 2019. https://www.who.int/emergencies/ten-threats-to-global-health-in-2019.

[2] European Centre for Disease Prevention and Control Measles. https://ecdc.europa.eu/en/measles.

[3] CBS News Measles Outbreak. https://www.cbsnews.com/news/measles-outbreak.

[4] The Strategic Advisory Group of Experts (SAGE) (2014). Report of the SAGE Working Group on Vaccine hesitancy. SAGE Rep..

[5] Larson H.J., De Figueiredo A., Xiahong Z., Schulz W.S., Verger P., Johnston I.G., Cook A.R., Jones N.S. (2016). EBioMedicine The State of Vaccine Confidence 2016: Global Insights Through a 67-Country Survey. EBIOM.

[6] Mac Donald N.E. (2015). Vaccine hesitancy: Definition, scope and determinants. Vaccine.

[7] Larson H.J., Brocard P. (2010). The India HPV-vaccine suspension. Lancet.

[8] Rosselli R., Martini M., Bragazzi N.L. (2016). The old and the new: Vaccine hesitancy in the era of the Web 2.0. Challenges and opportunities. J. Prev. Med. Hyg..

[9] McClure C.C., Cataldi J.R., O’Leary S.T. (2017). Vaccine Hesitancy: Where We Are and Where We Are Going. Clin. Ther..

[10] Tafuri S., Gallone M.S. (2014). Addressing the anti-vaccination movement and the role of HCWs. Vaccine.

[11] Jung M. (2018). Challenges of Vaccinations in the Era of New Media Communication. Health Care Manag..

[12] Holden J. (2010). Lancet retracts 12-year-old article linking autism to MMR. CMAJ.

[13] Rao T.S.S., Andrade C. (2011). The MMR vaccine and autism: Sensation, refutation, retraction, and fraud. Indian J. Psychiatry.

[14] Smith T.C. (2017). Vaccine Rejection and Hesitancy: A Review and Call to Action. Open Forum Infect. Dis..

[15] Peretti-Watel P., Larson H.J., Ward J.K., Schulz W.S., Verger P. (2015). Vaccine hesitancy: Clarifying a theoretical framework for an ambiguous notion. PLoS Curr..

[16] Larson H.J., Clarke R.M., Jarrett C., Eckersberger E., Levine Z., Schulz W.S., Paterson P. (2018). Measuring trust in vaccination: A systematic review. Hum. Vaccine Immunother..

[17] Shapiro G.K., Tatar O., Dube E., Amsel R., Knauper B., Naz A., Perez S., Rosberger Z. (2018). The vaccine hesitancy scale: Psychometric properties and validation. Vaccine.

[18] Larson H.J., Jarrett C., Schulz W.S., Chaudhuri M., Zhou Y., Dube E., Schuster M., MacDonald N.E., Wilson R. (2015). Measuring vaccine hesitancy: The development of a survey tool. Vaccine.

[19] Braun V., Clarke V. (2014). What can “thematic analysis” offer health and wellbeing researchers?. Int. J. Qual. Stud. Health Well-Being.

[20] Braun V., Clarke V. (2006). Using thematic analysis in psychology. Qual. Res. Psychol..

[21] Lombard M., Snyder-Duch J., Campanella Bracken C. (2017). Intercoder Reliability. The SAGE Encyclopedia of Communication Research Methods.

[22] Freelon D.G. (2010). ReCal: Intercoder Reliability Calculation as a Web Service. Int. J. Internet Sci..

[23] Viera A.J., Garrett J.M. (2005). Understanding Inter-observer Agreement: The Kappa Statistic. Fam. Med..

[24] Hallgren K.A. (2012). NIH Public Access. Tutor. Quant. Methods Psychol..

[25] Krippendorff K. (2004). Content Analysis: An Introduction to Its Methodology.

[26] Dubé E., Leask J., Wolff B., Hickler B., Balaban V., Hosein E., Habersaat K. (2017). The WHO Tailoring Immunization Programmes (TIP) approach: Review of implementation to date. Vaccine.

[27] Biosta TGV. https://biostatgv.sentiweb.fr/.

[28] VassarStats: Website for Statistical Computation. http://www.vassarstats.net/.

[29] Centrul National de Statistica pentru Boli Transmisibile. cnscbt.ro/index.php/rapoarte-anuale/.

[30] Murrero M., Rice R.E. (2013). The Internet and Health Care: Theory, Research and Practice.

[31] Van Laer J., Van Aelst P. (2010). Internet and social movement action repertoire: Opportunities and limitations. Inform. Commun. Soc..

[32] Betsch C. Dr. (2012). Jekyll or Mr. Hyde? (How) the Internet influences vaccination decisions: Recent evidence and tentative guidelines for online vaccine communication. Vaccine.

[33] Witteman H.O., Zikmund-Fisher B.J. (2012). The defining characteristics of Web 2.0 and their potential influence in the online vaccination debate. Vaccine.

[34] Ward J.K., Peretti-Watel P., Verger P. (2016). Vaccine criticism on the Internet: Propositions for future research. Hum. Vaccines Immunother..

[35] Kang G.J., Ewing-Nelson S.R., Mackey L., Schlitt J.T., Marathe A., Abbas K.M., Swarup S. (2017). Semantic network analysis of vaccine sentiment in online social media. Vaccine.

[36] Miton H., Mercier H. (2015). Science and Society Cognitive Obstacles to Pro-Vaccination Beliefs. Trends Cogn. Sci..

[37] Mcphetres J., Zuckerman M. (2018). Religiosity predicts negative attitudes towards science and lower levels of science literacy. PLoS ONE.

[38] Kata A. (2012). Anti-vaccine activists, Web 2.0, and the postmodern paradigm—An overview of tactics and tropes used online by the anti-vaccination movement. Vaccine.

[39] Slovic P., Finucane M.L., Peters E., Macgregor D.G. (2004). Risk as Analysis and Risk as Feelings: Some Thoughts about Affect, Reason, Risk, and Rationality. Risk Anal..

[40] Damasio A.R. (1994). Descartes’ Error: Emotion, Reason, and the Human Brain.

[41] Ropeik D. (2013). How society should respond to the risk of vaccine rejection. Hum. Vaccine Immunother..

[42] Karafillakis E., Dinca I., Apfel F., Cecconi S., Wűrz A., Takacs J., Suk J., Celentano L.P., Kramarz P., Larson H.J. (2016). Vaccine hesitancy among healthcare workers in Europe: A qualitative study. Vaccine.

[43] Karafillakis E., Larson H.J. (2018). The paradox of vaccine hesitancy among healthcare professionals. Clin. Microbiol. Infect..

[44] Agrinier N., Le Maréchal M., Fressard L., Verger P., Pulcini C. (2017). Discrepancies between general practitioners’ vaccination recommendations for their patients and practices for their children. Clin. Microbiol. Infect..

[45] Suryadevara M., Handel A., Bonville C.A., Cibula D.A., Domachowske J.B. (2015). Pediatric provider vaccine hesitancy: An under-recognized obstacle to immunizing children. Vaccine.

[46] Dubé E. (2017). Addressing vaccine hesitancy: The crucial role of healthcare providers. Clin. Microbiol. Infect..

[47] Van der Linden S.L., Clarke C.E., Maibach E.W. (2015). Highlighting consensus among medical scientists increases public support for vaccines: Evidence from a randomized experiment. BMC Public Health.

[48] Morariu S., Tarcea M., Moldovan H., Dobreanu M. (2016). Human Papillomavirus (HPV) Infection and HPV Vaccination: Assessing the Level of Knowledge among Students of the University of Medicine and Pharmacy of Tîrgu Mureş, Romania. Acta Dermatovenerol. Croat..

[49] Paterson P., Meurice F., Stanberry L.R., Glismann S., Rosenthal S.L., Larson H.J. (2016). Vaccine hesitancy and healthcare providers. Vaccine.

[50] Wilson R., Paterson P., Larson H.J. (2019). Strategies to improve maternal vaccination acceptance. BMC Public Health.

[51] Betsch C., Bödeker B., Schmid P., Wichmann O. (2018). How baby’s first shot determines the development of maternal attitudes towards vaccination. Vaccine.

[52] Mendel-Van Alstyne J.A., Nowak G.J., Aikin A.L. (2017). What is ‘confidence’ and what could affect it? A qualitative study of mothers who are hesitant about vaccines. Vaccine.

[53] Vanderslott S. (2019). Exploring the meaning of pro-vaccine activism across two countries. Soc. Sci. Med..

[54] (2018). WHO Regional Office: Tailoring Immunization Programmes (TIP) An Introductory Overview. https://www.who.int/immunization/programmes_systems/Global_TIP_overview_May2018.pdf.

[55] Jackson C., Bedford H., Cheater F.M., Condon L., Emslie C., Ireland L., Kemsley P., Kerr S., Lewis H.J., Mytton J. (2017). Needles, Jabs and Jags: A qualitative exploration of barriers and facilitators to child and adult immunisation uptake among Gypsies, Travellers and Roma. BMC Public Health.

[56] De Graaf P., Rotar Pavlič D., Zelko E., Vintges M., Willems S., Hanssens L. (2016). Primary care for the Roma in Europe: Position paper of the European forum for primary care. Zdr. Varst..

[57] de Figueiredo A., Larson H.J., Johnston I.G., Smith D.M., Jones N.S. (2016). Forecasted trends in vaccination coverage and correlations with socioeconomic factors: A global time-series analysis over 30 years. Lancet.

[58] Omer S.B., Salmon D.A., Orenstein W.A., Halsey N. (2009). Vaccine Refusal, Mandatory Immunization, and the Risks of Vaccine-Preventable Diseases. N. Engl. J. Med..

[59] Dubé E., Gagnon D., MacDonald N.E. (2015). Strategies intended to address vaccine hesitancy: Review of published reviews. Vaccine.

[60] Dubé E., Gagnon D., Nickels E., Jeram S., Schuster M. (2014). Mapping vaccine hesitancy-Country-specific characteristics of a global phenomenon. Vaccine.

[61] Eskola J., Duclos P., Schuster M., MacDonald N.E. (2015). How to deal with vaccine hesitancy?. Vaccine.

[62] Goldstein S., MacDonald N.E., Guirguis S. (2015). Health communication and vaccine hesitancy. Vaccine.

